# Anti-N-Methyl-D-Aspartate Receptor Encephalitis Associated With Small Cell Lung Cancer: A Case Report

**DOI:** 10.7759/cureus.78702

**Published:** 2025-02-07

**Authors:** Yemesrach Kerego, Colleen Bramwell, Bolanle Dada

**Affiliations:** 1 Research and Innovation, St. Luke's University Health Network, Bethlehem, USA; 2 Internal Medicine, St. Luke's University Health Network, Easton, USA

**Keywords:** anti-nmdar encephalitis, autoimmune encephalopathy, nmdar antibody encephalitis, paraneoplastic neurological syndromes, rare cause of altered mental status, small cell lung cancer (sclc)

## Abstract

Anti-N-methyl-d-aspartate receptor encephalitis (NMDARE) is an autoimmune disorder with a clinical presentation that overlaps with that of a myriad of neuropsychiatric conditions. Delays in diagnosis have been linked to long-term complications that affect a patient's quality of life.

A 60-year-old female patient with a medical history of emphysema with chronic respiratory failure, hypertension, diabetes, morbid obesity, and hyperlipidemia presented to the hospital after she was found confused at home with limited verbal output, raising concerns for cerebrovascular events or toxic metabolic encephalitis. Neuroimaging and EEG results were unremarkable. The patient continued to experience respiratory distress that delayed diagnostic procedures. She was later diagnosed with small-cell lung cancer, and cerebrospinal fluid tests were positive for anti-N-methyl-d-aspartate receptor antibodies. Gradual improvement in cognitive function and speech was noted after treatment with immunotherapy.

This case study underscores the importance of considering rare neurological conditions in patients with heightened cardiovascular risk profiles. Furthermore, it provides insights into the potential diagnostic and therapeutic challenges that can arise in the domain of anti-NMDARE in such patients.

## Introduction

N-methyl-d-aspartate receptor (NMDAR) is an inotropic glutamate receptor widely distributed throughout the brain. It is involved in synaptic transmission and neuronal plasticity. Anti-NMDAR encephalitis (NMDARE) is an autoimmune disorder characterized by antibodies targeting NMDAR [1]. Owing to different NMDAR epitopes, the condition is marked by diverse clinical presentations, such as neurologic deficits, cognitive impairment, psychiatric symptoms, movement disorders, and seizure disorders.

The triggers for this autoimmune response remain unknown. Still, a growing number of case reports have linked anti-NMDARE to viral infections, neoplasms (especially ovarian teratomas), small-cell lung carcinoma (SCLC), and other autoimmune disorders. Although the condition is self-limiting, delay in diagnosis has the potential to result in a severe form with psychiatric, cognitive, and neurologic sequelae [1-3].

We present a case of anti-NMDARE in a patient with a history of multiple cardiovascular risk factors who presented to the hospital with an altered mental status and limited verbal output. Despite the initial consideration of cerebrovascular events or toxic metabolic encephalitis, a comprehensive and detailed evaluation revealed anti-NMDARE, prompting initiation of intravenous immunoglobulin (IVIG) and steroids, which led to the gradual resolution of symptoms.

This article was previously presented as a meeting abstract at the 2024 Academic International Medicine Annual Conference on June 22, 2024.

## Case presentation

A 60-year-old female patient with a history of hypertension, type II diabetes, hyperlipidemia, obstructive sleep apnea, emphysema, and chronic respiratory failure (on 3 L of O_2_ via nasal cannula at home) presented to the hospital with an altered mental status after her son found her confused without oxygen. The patient was reported to be well two days before the presentation. Her medications included albuterol as needed, fluticasone-salmeterol inhaler, lisinopril, metformin, glimepiride, atorvastatin, and pregabalin.

Physical examination was pertinent for morbid obesity (BMI 44.98 kg/m^2^), confusion, flat affect, delayed responses, and limited verbal output. The results of cranial nerves and sensory examinations were normal. Motor examination revealed limited movement of the lower extremities. Given her mental status and aphasia with multiple comorbidities, cerebrovascular events were initially considered. The stroke pathway was followed. Routine laboratory tests, echocardiography, ECG, computed tomography (CT) of the head without contrast, and brain MRI were nonrevealing. Although the initial EEG was normal, a follow-up EEG revealed slowing of the posteriorly dominant rhythm and intermittent generalized mixed-frequency theta activity, suggesting mild nonspecific diffuse cerebral dysfunction. CT of the chest revealed a right upper pulmonary mass suspected to be malignant. The patient had a 40-pack-year history of smoking, and screening chest CT scans were performed for lung cancer during her follow-ups, but the results were negative.

The patient was transferred to the ICU on the fifth day of admission due to hypoxia (oxygen saturation of 86% on a high-flow nasal cannula at 60 L/minute, 100% oxygen), raising concerns about toxic metabolic encephalopathy. She was initially placed on bilevel positive airway pressure (BiPAP) 16/6; however, following continued oxygen demand and persistent hypoxemia, arterial blood gas analysis: pH 7.465 (normal: 7.35-7.45), partial pressure of carbon dioxide 38.3 mm Hg (normal: 35-45 mm Hg), partial pressure of oxygen 58.3 mm Hg (normal 80-100 mm Hg), and bicarbonate 26.9 mmol/L (normal: 22-28 mmol/L), the patient was intubated and mechanically ventilated for five days. On the day of intubation, septic shock developed, with a pulse rate of 112 bpm, a temperature of 101.3°F (38.5°C), a respiratory rate of 29, white blood cell (WBC) count at 18.02 × 10³/μL (normal: 4.5-11.0 × 10³/μL), chest radiography indicating right lower lung pneumonia, and bronchial culture identifying *Serratia marcescens*. Intravenous (IV) cefepime was initiated.

In light of the respiratory status and septic shock, lumbar puncture (LP) and endoscopic bronchial ultrasound (EBUS) biopsy were deferred despite the high suspicion of autoimmune or paraneoplastic etiology after chest CT. Postextubation, LP was performed, and cerebrospinal fluid (CSF) analysis indicated a positive NMDAR antibody. The patient had recurrent low-grade fever with a peak temperature of 101.3°F (38.5°C). Following extensive work-up, no alternative cause was identified, and the fever was attributed to the underlying malignancy.

Diagnostic assessment

**Figure 1:**
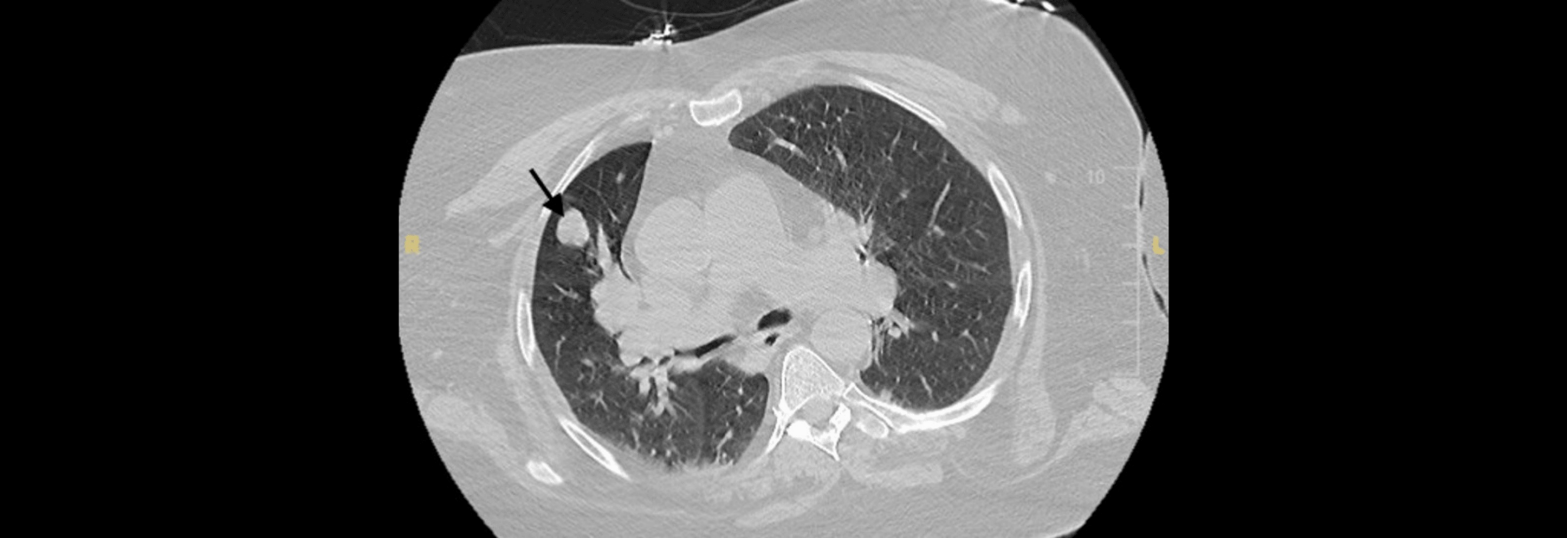
CT of the chest showing right upper lobe nodule (arrow) CT: computed tomography

Routine lab tests were within the normal limit, including complete blood count, urinalysis, and complete metabolic profile. HgA1c was 6.6 (target range for diabetics: <7), and lipid profile showed triglycerides 84 mg/dL (normal: <150 mg/dL), low-density lipoprotein 67 mg/dL (normal: <100 mg/dL), and high-density lipoprotein 29 mg/dL (normal: ≥50 mg/dL for women). Initial neuroimaging studies, including a noncontrast head CT and contrast MRI of the brain, showed no acute abnormalities. A CT angiogram of the head and neck ruled out vascular causes. CT of the chest identified a 1.8-cm left upper lobe nodule with marked hilar adenopathy, consistent with malignancy (Figure 1). Workup for distant metastasis was negative. Following stability in the patient’s respiratory status, an LP was performed with CSF analysis positive for NMDAR antibodies and elevated lymphocytes (Table 1). EBUS biopsy and further histological and immunohistochemical analysis confirmed the presence of SCLC. A CT scan of the abdomen and pelvis performed to rule out teratomas was negative.

**Table 1:** Results of CSF study CSF: cerebrospinal fluid; IgG: immunoglobulin G; NA: not assessed; NMDA-R: N-methyl D-aspartate receptor

Parameters	Value	Normal range
Gross appearance	Clear	Clear
Total nucleated cells	100/μL	0-5/μL
Red blood cells	1/μL	0-10/μL
Lymphocytes	95%	28-96%
Monocytes	5%	0-30%
Total protein, CSF	62 mg/dL	15-45 mg/dL
Glucose, CSF	100 mg/dL	40-70 mg/dL
Oligoclonal bands	NA	Absent
IgG index	NA	0.28-0.6
NMDA-R antibody, CSF	Positive, 1:10	Negative, <1:10
Other autoimmune markers	Negative	Negative
Meningitis/encephalitis panel	Negative for all pathogens	Negative
Cytology	Negative	Negative

Therapeutic intervention

The patient initially underwent assessment and management under the stroke pathway, which included aspirin, CNS monitoring, and close monitoring of blood pressure and glucose levels. This pathway was discontinued after the diagnosis of NMDARE. Precautions were applied to avoid sedatives. Occupational, physical, and speech therapy were initiated upon presentation and continued throughout the patient’s hospitalization. Respiratory support was provided depending on demand, initially through high-flow nasal oxygen, followed by BiPAP, endotracheal intubation with subsequent reintroduction of BiPAP, and eventual weaning off oxygen support. Despite completing a seven-day course of antibiotics and interval radiographic improvement and normalization of WBCs, she continued to have neurologic dysfunction and increased oxygen requirement. After excluding other possible causes, anti-NMDARE was considered. She was started on a five-day course of IVIG 400 mg/kg every 24 hours and 1,000-mg IV methylprednisolone per protocol, and she showed significant improvement. Notably, the patient’s fasting blood glucose averaged 246 mg/dL following the initiation of steroid therapy, requiring a hold on home medications (metformin and glimepiride) and initiation of an insulin drip. Otherwise, medications were well tolerated.

Follow-up and outcome

Following treatment, the patient gradually improved in cognitive and physical functions. By the time of discharge, she was oriented, was able to follow commands, and showed marked improvement in verbal output and motor function. She was discharged with plans for comprehensive follow-up with physical and speech therapy, hematology-oncology, primary care providers, and pulmonology teams. The chemotherapy regimen for SCLC was scheduled to begin after discharge. Progress in physical rehabilitation and cognition is closely monitored through regular follow-up.

## Discussion

Owing to the diverse topographical distribution of NMDARs within the CNS, encompassing regions such as the hippocampus, cortex, and basal ganglia, anti-NMDARE has a broad spectrum of clinical manifestations [2,4]. This syndrome typically progresses through several phases. It often begins with a prodromal phase with flu-like symptoms, progressing to the psychiatric phase with symptoms such as hallucinations, confusion, and mood disturbances, and then transitions to the neurologic phase with seizures, movement disorders, and impaired consciousness, followed by a dysautonomic phase with hypoventilation, temperature dysregulation, and cardiovascular instability, occasionally requiring pacemakers [2,4-6].

Studies have shown that over three-quarters of patients diagnosed with anti-NMDARE, even those with associated SCLC, initially present with psychiatric features such as hallucinations, depression, anxiety, and agitation, frequently leading to misdiagnosis of schizophrenia or mood disorders [2,4-6]. However, in the absence of dominant psychiatric features and the presence of altered mentation, aphasia, and a high cardiovascular risk profile, a primary consideration of cerebrovascular events, such as stroke, is reasonable, as in our case. Although respiratory failure and prolonged hypoxia have the potential to reduce cognitive reserve, a positive NMDAR antibody and the absence of improvement with supportive measures make the diagnosis of toxic metabolic encephalopathy less likely. Additionally, despite a continued record of low-grade fever, negative blood cultures and CSF studies ruled out infectious encephalitis.

The definitive diagnosis of anti-NMDARE is established by the presence of NMDAR antibodies in either the CSF or serum, with CSF demonstrating higher sensitivity and specificity [2,5,6]. In our case, there was a high suspicion of an autoimmune or paraneoplastic etiology in relation to the lung mass. However, concurrent septic shock and respiratory status requiring intubation necessitated the prioritization of supportive measures, delaying diagnostic procedures such as LP and EBUS. Other less-specific CSF characteristics observed in anti-NMDARE include mild lymphocytic pleocytosis and mildly elevated protein and oligoclonal bands [6]. EEG is a particularly important test with 90% sensitivity and often displays nonspecific generalized slowing [2]. Initial EEG readings, as in our patient, can be normal and require repeated testing. The recommended treatment for anti-NMDARE includes a five-day course of IV methylprednisolone (1 g/day) and IV plasmapheresis or IVIG (0.4 g/kg/day). Weekly rituximab or monthly cyclophosphamide can be used as second-line therapy [2,5,7].

Anti-NMDARE is a self-limiting inflammatory condition characterized by a slow recovery trajectory, often necessitating prolonged rehabilitation with support from occupational, physical, and speech therapies [2,5,6]. Approximately a quarter of individuals with this condition experience persistent neurological and cognitive deficits [5]. While milder symptoms and better outcomes are reported with ovarian teratomas, higher mortality is reported in cases of delayed diagnosis and those associated with other neoplasms such as SCLC [3,4]. Early initiation of immunosuppressants has been shown to reduce the risk of relapse. A 12-24% risk of relapse exists, particularly among patients without detectable tumors, reinforcing the importance of prolonged immunosuppressive therapy in such patients [2,3,5,8]. A search for teratomas using abdominal and pelvic imaging was conducted on our patient, which aligns with the recommendations [2,3,5]. Furthermore, antibody titers are directly related to the severity of the disease and have been noted to decline with the improvement of symptoms; hence, they can be utilized for follow-up assessments [2,3,5].

## Conclusions

We report a rare case of anti-NMDARE with positive CSF NMDAR antibodies in a patient who presented to the hospital with altered mentation and was later diagnosed with small-cell lung cancer. This study underscores the importance of considering rare neurological conditions in patients with a multifactorial cardiovascular risk profile. By emphasizing the need for a comprehensive evaluation in managing patients presenting with altered mentation, this case study provides insight into potential diagnostic and therapeutic challenges that can arise in the domain of NMDARE in patients with multiple comorbidities.
